# Minimal impact of beam projection angle deviations in skyline (Laurin) view and the efficacy of the anterior border of proximal tibia as a guiding landmark

**DOI:** 10.1007/s00256-024-04619-1

**Published:** 2024-02-13

**Authors:** Sung Eun Kim, Sunghyun Hwang, Ji Han Lee, Geunwu Gimm, Hyuk-Soo Han, Byung Sun Choi, Du Hyun Ro

**Affiliations:** 1 Department of Orthopaedic Surgery, Seoul National University Hospital, 101 Daehak-Ro, Jongno-Gu, Seoul, 110-744 South Korea; 2 Department of Orthopaedic Surgery, Seoul National University College of Medicine, Seoul, South Korea; 3 Department of Biomedical Engineering, Seoul National University College of Medicine, Seoul, South Korea; 4 CONNECTEVE Co., Ltd, Seoul, South Korea

**Keywords:** Skyline view, Laurin view, Beam projection angle, Anterior border of proximal tibia, Deviation

## Abstract

**Objective:**

Obtaining an optimal knee skyline view is challenging due to inaccuracies in beam projection angles (BPAs) and soft tissue obscuring bony landmarks. This study aimed to assess the impact of BPA deviations on patellofemoral index measurements and assessed the anterior border of the proximal tibia as an anatomic landmark for guiding BPAs.

**Materials and methods:**

This retrospective study consisted of three parts. The first was a simulation study using 52 CT scans of knees with a 20° flexion contracture to replicate the skyline (Laurin) view. Digitally reconstructed radiographs simulated neutral, 5° downward, and 5° upward tilt BPAs. Five patellofemoral indices (sulcus angle, congruence angle, patellar tilt angle, lateral facet angle, and bisect ratio) were measured and compared. The second part was a proof of concept study on 162 knees to examine patellar indices differences across these BPAs. Lastly, the alignment of the anterior border of the proximal tibia with the BPA tangential to the patellar articular surface was tested from the CT scans.

**Results:**

No significant differences in patellofemoral indices were found across various BPAs in both the simulation and proof of concept studies (all *p* > 0.05). The angle between the anterior border of the proximal tibia and the patellar articular surface was 1.5 ± 5.3°, a statistically significant (*p* = 0.037) yet clinically acceptable deviation.

**Conclusion:**

Patellofemoral indices in skyline view remained consistent regardless of BPA deviations. The anterior border of the proximal tibia proved to be an effective landmark for accurate beam projection.

## Introduction

Evaluation of the patellofemoral joint is essential for determining the severity of knee arthritis, patellar subluxation/dislocation, and trochlear dysplasia. Various radiographic techniques, such as the Laurin, Merchant, and Hughston views, are methods to obtain the knee skyline (axial) view for this purpose [1–3]. Several patellofemoral indices are measured in these views to detect patellofemoral abnormalities. Although there is no gold standard for the skyline view due to the unique advantages and limitations of each technique and variations in clinical settings across healthcare institutions [4], prior research suggests that a knee flexion angle between 20 and 30° optimizes evaluation and ensures consistent reproducibility of patellofemoral indices [2, 3, 5].

Challenges in obtaining an optimal skyline image arise from factors such as inaccuracies in the beam projection angle (BPA), patient knee flexion angle errors, and knee deformities. The image accuracy may be further compromised by soft tissue that obscures bone positioning, potentially leading to BPA errors and resulting in images with double lines and blurred contours of the patella and femur. Consequently, the precision of the skyline view largely depends on the radiographer’s expertise, often leading to inconsistent outcomes due to human error [6]. Moreover, concerns about radiation exposure and time constraints in high-volume clinical settings become pronounced when repetition of X-rays is necessary to achieve an accurate patellofemoral joint view. Therefore, if patellofemoral indices were consistent despite BPA errors, it would reduce the time spent repeating the X-ray to obtain the perfect skyline view. Also, identifying an anatomic landmark to guide the desired BPA may be beneficial.

Thus, the primary objectives of this study were (1) to evaluate the consistency of patellofemoral indices among possible BPA errors via a simulation study and (2) to verify this consistency by obtaining real patient data. The secondary objective was to identify an anatomical landmark that aids in determining the optimal BPA for knee skyline views.

## Materials and methods

**Fig. 1:**
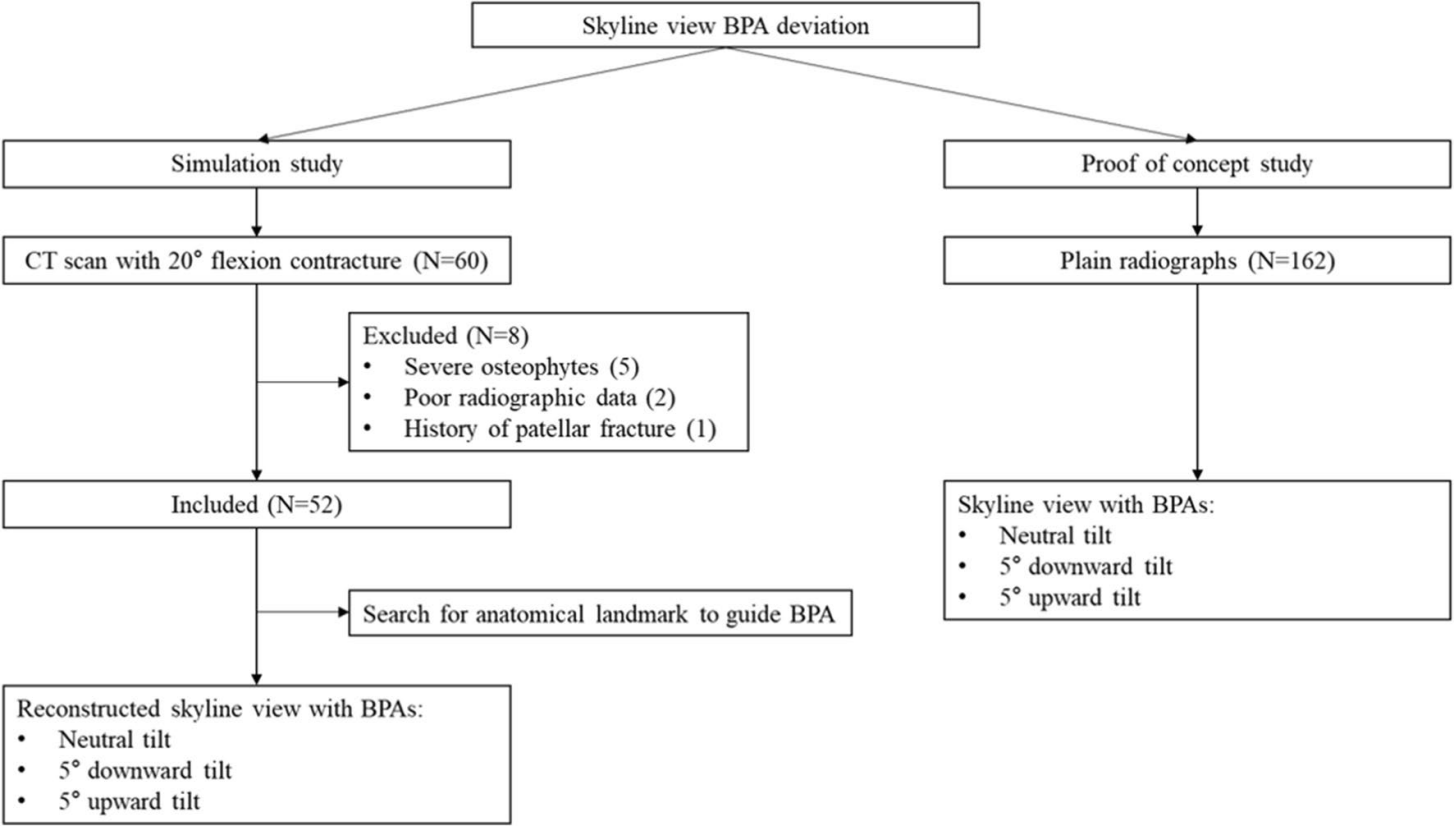
Flow chart of the study. BPA, beam projection angle

This retrospective study was approved by the Institutional Review Board (IRB) of the authors’ institute (IRB No: 2312–057-1491), with the requirement for informed consent waived. The study was conducted in three parts: the first involved simulating BPAs for skyline views using computed tomography (CT) scans and digitally reconstructing images. The second part applied these findings to real patient X-ray data to validate the results from the simulation study. In the third part, the search for an anatomical landmark to guide the BPA was conducted, using the CT scans from the first part (Fig. 1). Demographic factors including age, sex, body mass index, and patellar morphology (Wiberg classification) were collected [7].

### Patellofemoral indices according to BPA errors (CT simulation study)

CT scans (SOMATOM Force, Siemens Healthineers, Erlangen, Germany) from October 2016 to February 2021 were analyzed for patients admitted for total knee arthroplasty with a 20° flexion contracture. This particular patient group was chosen to replicate the Laurin view in CT scans, in line with the preoperative Laurin view radiographs taken at the authors’ institute. The exclusion criteria were poor radiographic data, measurement difficulties arising from severe osteophytes, and a history of patellar fracture or patellar subluxation/dislocation. Initially, 60 knees with a 20° flexion contracture were reviewed, but eight were excluded (five due to severe osteophytes, two due to poor radiographic data, and one due to a history of patellar fracture), leaving 52 knees in the study.

**Fig. 2:**
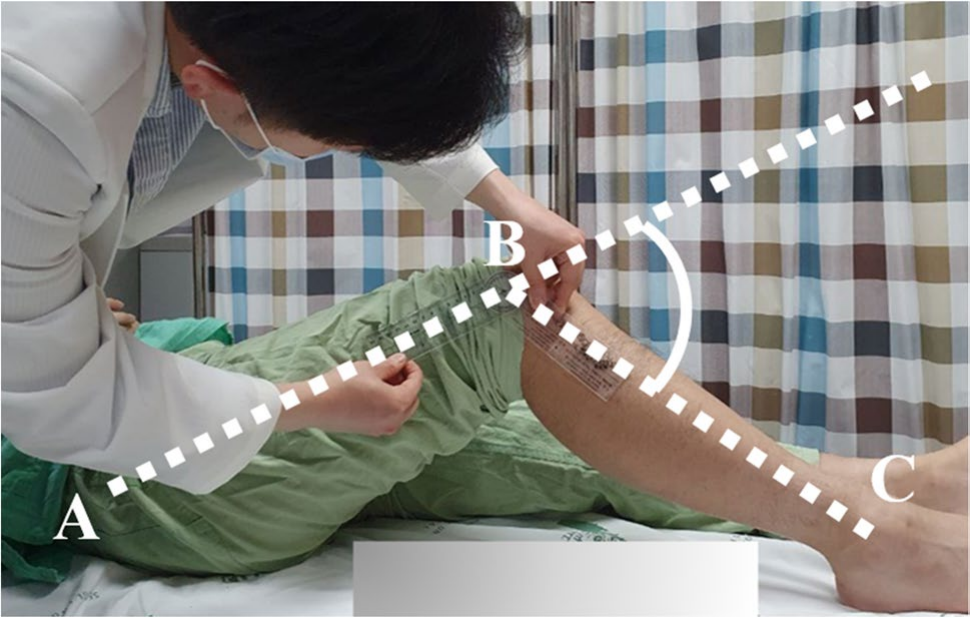
Measurement of knee flexion contracture. The degree of knee flexion contracture is determined by measuring the angle formed between line AB and line BC. A: Greater trochanter of femur. B: Lateral epicondyle of femur. C: lateral malleolus of the ankle

**Fig. 3:**
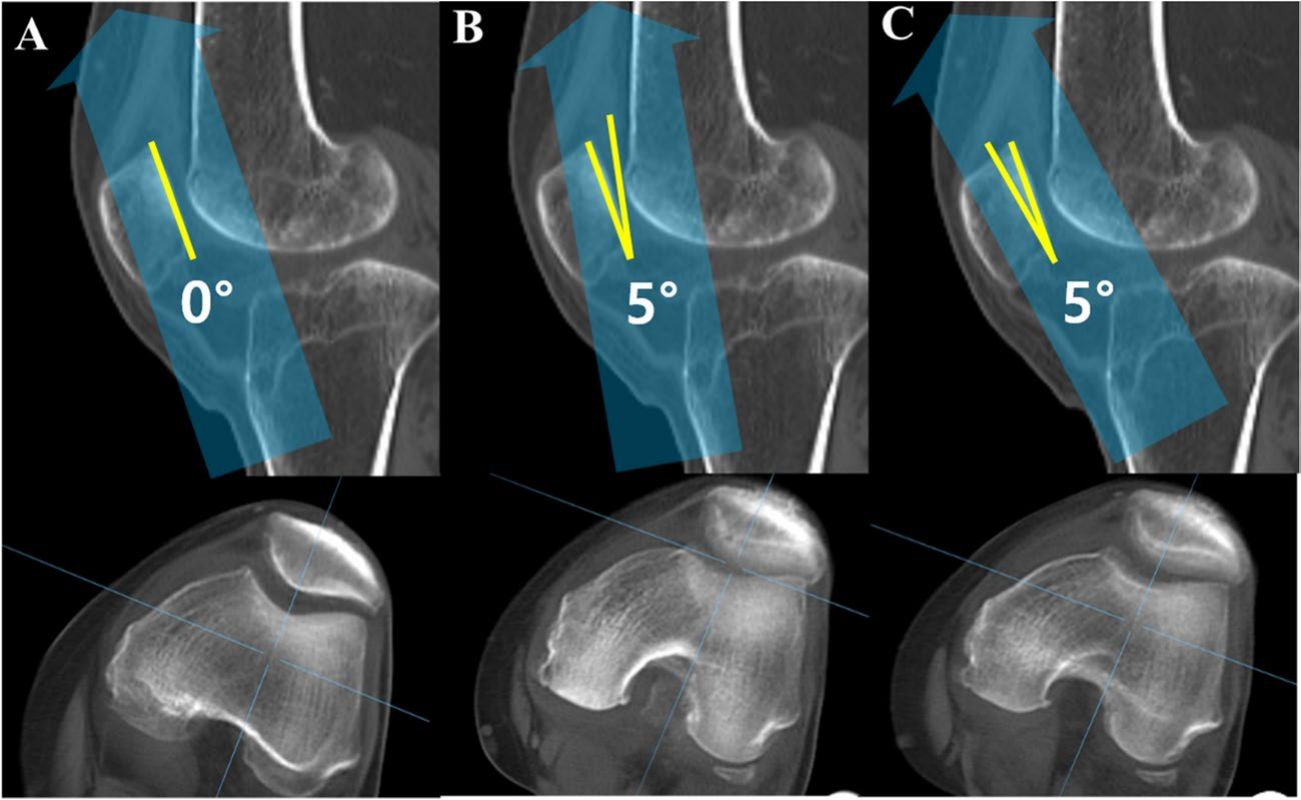
Examples of beam projection angle deviations and corresponding digitally reconstructed skyline images from CT scans. **A** Neutral tilt, **B** 5° downward tilt, and **C** 5° upward tilt. Blue arrows in each image indicate the direction of the beam projection, while yellow lines represent the patellar articular surface and the respective beam projection angle. As the beam projection angles deviate from the neutral tilt, there is a noticeable blurring of the patellar articular surface

Knee flexion contracture was measured in admitted patients in the supine position and was asked to fully straighten (extend) the knee. The degree of flexion contracture was measured by palpating the greater trochanter, lateral epicondyle of the femur, and lateral malleolus of the ankle, and measuring the angle between these three landmarks using a goniometer (Fig. 2). High-resolution CT scans were taken in the supine position with a sustained 20° flexion contracture, accessed via the Picture Archiving and Communication System (PACS) using INFINITT PACS M6 software (INFINITT Healthcare, Seoul, Korea). Digitally reconstructed radiographs simulated three different BPAs: neutral tilt (parallel to the patellar articular surface), 5° downward tilt, and 5° upward tilt (Fig. 3). The variation of 5° in either direction was intended to represent potential errors in BPAs. For angle variations exceeding 10°, measurements of patellofemoral indices were not feasible due to increased bony overlap and decreased image resolution.

**Fig. 4:**
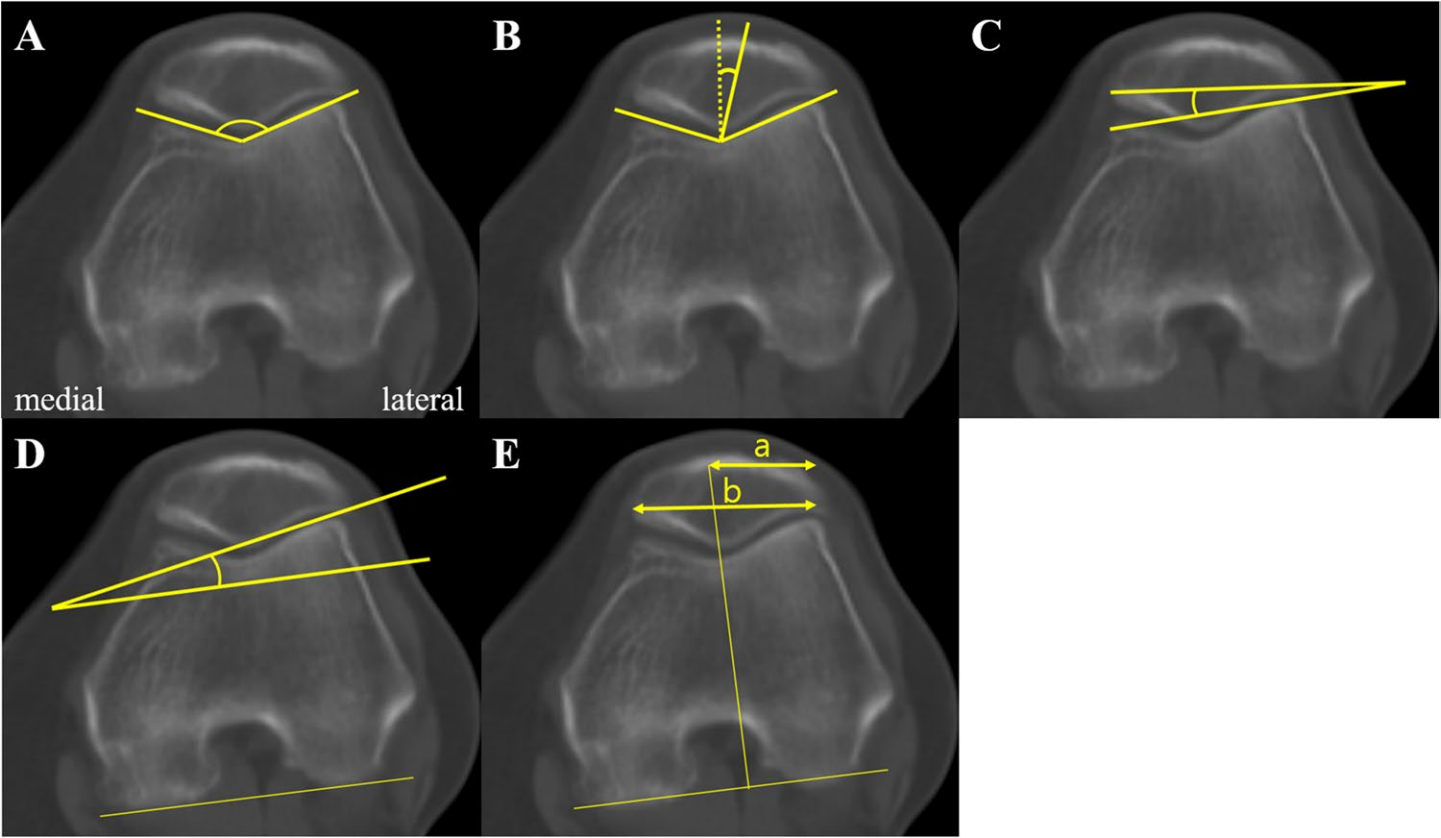
Measurement methods of patellofemoral indices. **A** Sulcus angle, **B** congruence angle, **C** patellar tilt, **D** lateral facet angle, and **E** bisect offset. In image **B**, the dotted line bisects the sulcus angle. In image **E**, the bisect offset is calculated as the ratio a/b

Five patellofemoral indices — sulcus angle, congruence angle, patellar tilt angle, lateral facet angle, and bisect offset — were evaluated according to the BPA variation. The measurement methodologies for these indices were as follows (Fig. 4): The sulcus angle was measured between lines drawn from the deepest point of the trochlear groove to the highest points on the medial and lateral femoral condyles [2]. The congruence angle was measured between the line bisecting the sulcus angle and the line connecting the deepest point of the trochlear groove to the lowest point of the patellar ridge [3]. The patellar tilt angle was measured by a line connecting the medial and lateral edges of the patella and the anterior intercondylar line of the femur [8]. The lateral facet angle was measured by the lateral patellar facet and a line parallel to the posterior femoral condyles [2]. The bisect offset was calculated by measuring the width of the patella and the lateral portion of the bisected width of the patella, with the bisecting line passing through the deepest point of the trochlear groove and being perpendicular to the posterior femoral condyles [9].

### Patellofemoral indices according to different BPAs (proof of concept study)

**Fig. 5:**
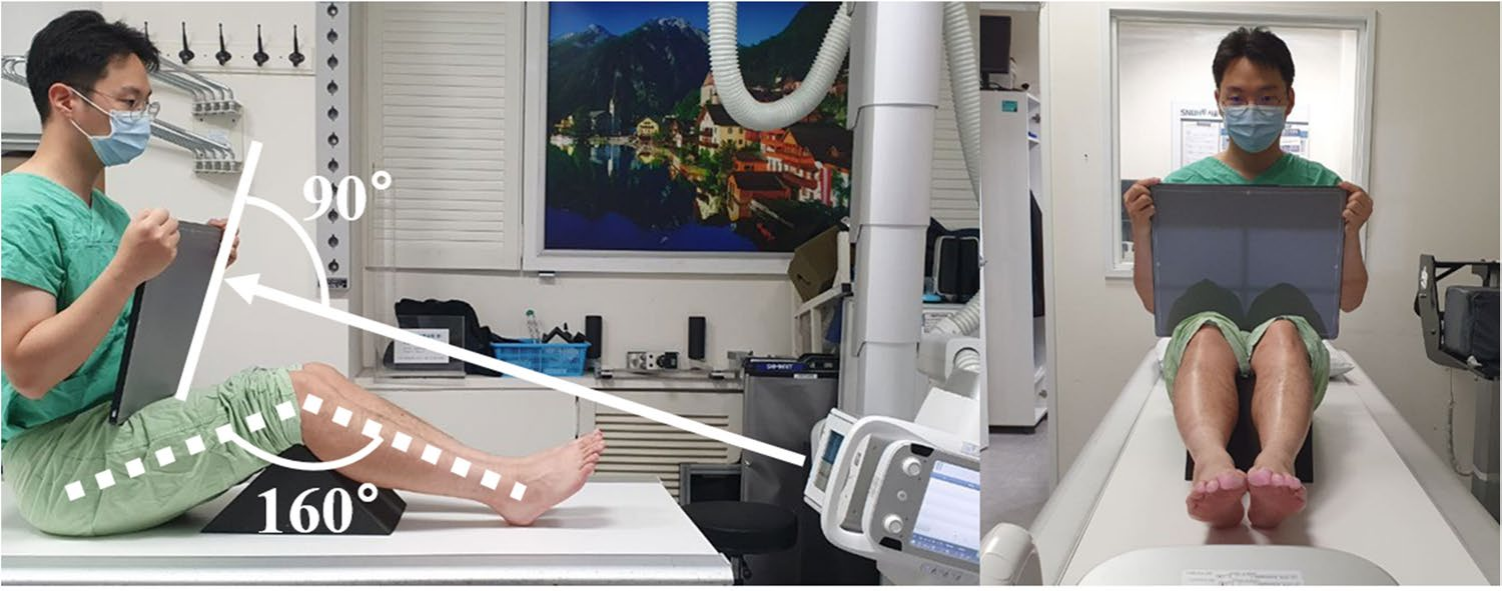
Example of obtaining a skyline (Laurin) view

**Fig. 6:**
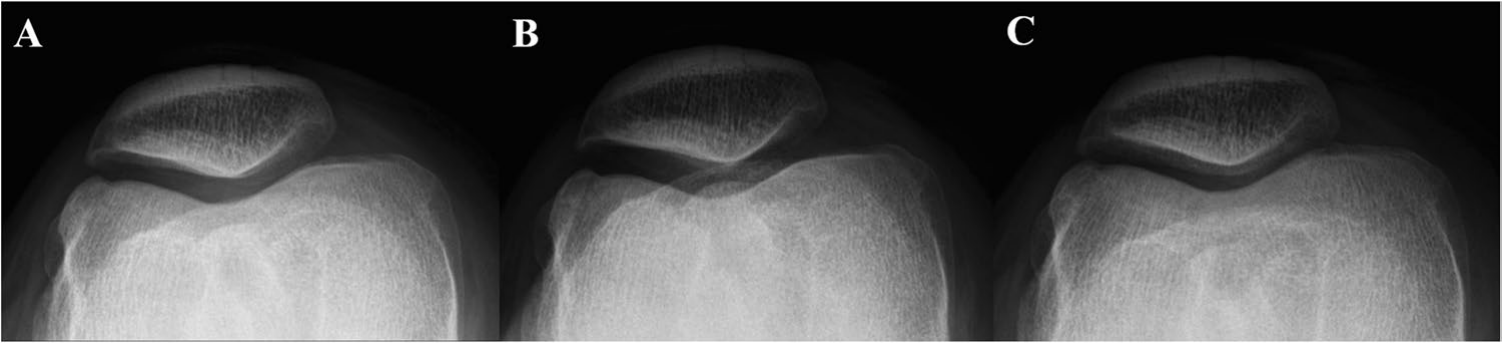
Examples of radiographs with beam projection angle deviations. **A** Neutral tilt, **B** 5° downward tilt, and **C** 5° upward tilt

In this phase of the study, patellofemoral indices (sulcus angle, congruence angle, and patellar tilt angle) were evaluated from patients who visited the outpatient clinic between May 2023 and July 2023. A total of 162 knees from 81 patients were analyzed, each having undergone three different knee skyline (Laurin view) radiographs with neutral tilt, 5° downward tilt, and 5° upward tilt. For the radiographs, patients were seated with a pillow placed beneath the knee to achieve a 20° knee flexion. The X-ray image detector was held by the patient perpendicular to the BPA, and the beam was directed from the distal to the proximal side, parallel to the lower leg (Fig. 5). After obtaining the neutral tilt radiograph, patients were asked to hold their position and the image detector as steady as possible, while the tube’s angulation was adjusted by 5° to acquire radiographs with 5° downward and upward tilts (Fig. 6). The lateral facet angles and bisect offset were not measured due to the lack of visualization of the posterior cortices of the femur, unlike in the simulation study where the entire femur contour was visible.

### Anatomic landmarks for determining BPA

**Fig. 7:**
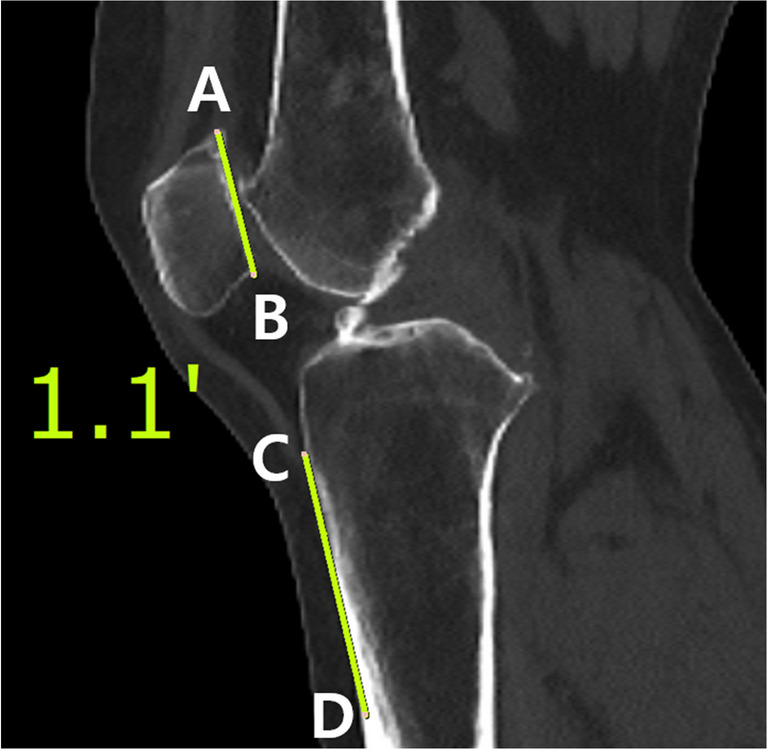
Measurement of the angle between the patellar articular surface and the anterior tibial border. Line AB represents the patellar articular surface, extending from the superior pole of the patella (point A) to the inferior pole of the patella (point B). Line CD represents the anterior tibial border, drawn from the tip of the tibial tuberosity (point C) to the proximal one-third of the anterior tibial cortex (point D)

The identification of anatomic landmarks to determine a BPA parallel to the patellar articular surface was conducted using the same CT scans of the 52 knees in the simulation study. On the sagittal cut of each CT scan, two key points were selected for analysis. The first point was the tip of the tibial tuberosity, presumed to be the most palpable landmark for the examiner. The second point identified was the proximal one-third of the anterior tibial cortex. A line connecting these two points, defined as the “Anterior border of proximal tibia,” was posited to represent the angle at which the beam projection targets the knee [10]. The angle between the anterior border of the proximal tibia and the line tangent to the patellar articular surface were then measured (Fig. 7). An angle of 0° was considered indicative of an optimal Laurin view. Additionally, the percentage of deviation beyond an absolute value of 5° from the optimal Laurin view was assessed.

### Data analysis

Paired sample *t*-tests were used to assess significant differences in patellofemoral indices among groups with neutral tilt, 5° downward tilt, and 5° upward tilt. The radiographic measurements were performed by two independent observers, both specializing in orthopedic surgery: one with over 7 years of expertise and the other an orthopedic surgeon with 3 years of experience. Both observers were blinded to the BPAs. Inter-observer reliability between the two surgeons, as well as intra-observer reliability for the senior surgeon, was assessed with a 4-week interval between measurements. For the statistical analysis, the measurements made by the more experienced orthopedic specialist were used. Power analysis for the paired sample *t*-tests, setting an alpha error at 0.05 and aiming for a power of 0.95, determined that a minimum sample size of 50 knees was necessary. Statistical analyses were performed using IBM SPSS Statistics 25 (IBM, Chicago, IL, USA) and Python 3.12.0, while power analysis was conducted using G*Power software 3.1.9.4 (Heinrich-Heine-Universität Düsseldorf, Düsseldorf, Germany).

## Results

Table 1 presents the demographic characteristics of the study participants. Patients in the simulation study were predominantly older and mostly women, with a higher prevalence of Wiberg classification type III and a lower prevalence of type I, in comparison to those in the proof of concept study. The radiographic measurements demonstrated strong inter-observer and intra-observer reliability, with intraclass correlation coefficients over 0.8 (Table 2).

**Table 1:** Demographic characteristics of the study patients

	Simulation study (*N* = 52)	Proof of concept study (*N* = 162)
Age (years)	71.8 ± 6.5 (46 to 84)	65.0 ± 15.7 (16 to 87)
Sex (women%)	94.3%	66.7%
Body mass index (kg/m^2^)	27.4 ± 3.8 (20.6 to 36.4)	25.0 ± 3.0 (19.4 to 33.7)
Wiberg classification		
Type I (%)	17.3%	28.4%
Type II (%)	53.9%	53.7%
Type III (%)	28.8%	17.9%

Data are presented in means ± standard deviations (range)

**Table 2:** Interobserver and intraobserver reliabilities of the patellofemoral indices measurements

	Sulcus angle	Congruence angle	Patellar tilt angle	Lateral facet angle	Bisect offset (%)
Interobserver ICC	0.943	0.887	0.902	0.891	0.803
Intraobserver ICC	0.971	0.897	0.906	0.956	0.874

*ICC* intraclass correlation coefficient

### Patellofemoral indices according to BPA errors (CT simulation study)

Table 3 displays the patellar indices across three different BPAs simulated in CT scans. No significant differences were observed in sulcus angle, congruence angle, patellar tilt angle, lateral facet angle, and bisect ratio between the neutral tilt, 5° downward tilt, and 5° upward tilt groups (all *p* > 0.05, Table 4).

**Table 3:** Patellofemoral indices of the 52 knees according to various beam projection angles (simulation study)

	Neutral tilt	5° downward tilt	5° upward tilt
Sulcus angle	133.9 ± 8.5 (115.1 to 151.4)	133.1 ± 8.0 (111 to 150)	133.8 ± 9.9 (113 to 168)
Congruence angle	4.1 ± 17.7 (− 43.5 to 42.0)	5.4 ± 17.8 (− 54.1 to 35.0)	5.3 ± 14.5 (− 30.3 to 35.0)
Patellar tilt angle	6.8 ± 3.2 (− 1.5 to 13.2)	7.0 ± 4.1 (− 0.8 to 16.1)	7.3 ± 4.1 (− 1.0 to 27.3)
Lateral facet angle	20.9 ± 5.8 (6.2 to 33.7)	21.7 ± 6.8 (4.1 to 40.3)	22.0 ± 6.9 (5.7 to 36.2)
Bisect offset (%)	61.7 ± 7.6 (34.6 to 80.4)	61.3 ± 6.9 (41.5 to 78.2)	62.2 ± 7.4 (42.4 to 79.0)

Data are presented in means ± standard deviations (range)

**Table 4:** Comparison of patellofemoral indices of the 52 knees according to various beam projection angles (simulation study)

	Sulcus angle	Congruence angle	Patellar tilt angle	Lateral facet angle	Bisect offset (%)
Neutral vs. 5° downward tilt	*p* = 0.454	*p* = 0.339	*p* = 0.831	*p* = 0.196	*p* = 0.509
Neutral vs. 5° upward tilt	*p* = 0.920	*p* = 0.759	*p* = 0.297	*p* = 0.066	*p* = 0.606
5° downward vs. 5° upward tilt	*p* = 0.515	*p* = 0.201	*p* = 0.428	*p* = 0.668	*p* = 0.300

### Patellofemoral indices according to different BPAs (proof of concept study)

Table 5 displays the patellofemoral indices across three different BPAs in skyline views. No significant differences were observed in sulcus angle, congruence angle, and patellar tilt angle, between the neutral tilt, 5° downward tilt, and 5° upward tilt groups (all *p* > 0.05, Table 6).

**Table 5:** Patellofemoral indices of the 162 knees according to various beam projection angles (proof of concept study)

	Neutral tilt	5° downward tilt	5° upward tilt
Sulcus angle	138.5 ± 5.4 (121.2 to 152.7)	138.7 ± 5.2 (121.5 to 149.1)	138.4 ± 5.1 (117.7 to 148.3)
Congruence angle	− 6.2 ± 16.6 (− 34.6 to 74.2)	− 6.4 ± 16.3 (− 47.0 to 61.6)	− 7.7 ± 17.4 (− 43.4 to 70.0)
Patellar tilt angle	5.0 ± 4.1 (− 5.6 to 16.5)	5.2 ± 4.2 (− 5.3 to 15.6)	4.9 ± 4.3 (− 5.2 to 19.3)

Data are presented in means ± standard deviations (range)

**Table 6:** Comparison of patellofemoral indices of the 162 knees according to various beam projection angles (proof of concept study)

	Sulcus angle	Congruence angle	Patellar tilt angle
Neutral vs. 5° downward tilt	*p* = 0.759	*p* = 0.304	*p* = 0.299
Neutral vs. 5° upward tilt	*p* = 0.134	*p* = 0.480	*p* = 0.497
5° downward vs. 5° upward tilt	*p* = 0.216	*p* = 0.088	*p* = 0.190

### Anatomic landmarks for determining BPA

The measured angle between the anterior border of the proximal tibia and the patellar articular surface was 1.5 ± 5.3°. This angle was statistically different from the ideal BPA of 0° (*p* = 0.037), yet was considered clinically acceptable. Only 19% of the angles deviated more than 5° from the perfect BPA, suggesting a high probability of achieving the Laurin view when using the anterior border of the proximal tibia as a reference.

## Discussion

The most important finding of this study is that a 5° variation in BPA during skyline view imaging does not significantly affect the measurement of patellofemoral indices, including congruence angle, patellar tilt angle, lateral facet angle, and bisect offset. This observation was consistent even in images presenting double lines and blurred contours of the patella and femur. In addition, the anterior border of the proximal tibia proved to be an effective landmark for directing the BPA. These results suggest that the anterior border of the proximal tibia can serve as a reliable reference for the skyline view, especially when the precision of beam projection is uncertain, with deviations up to 5° being tolerable.

Accurate beam projection is important in radiographic imaging, as parallax errors caused by deviations in BPA can lead to measurement inaccuracies around the knee [11]. The usefulness of various knee positioning and beam projection methods in the patellofemoral joint has been widely studied [1–3, 5, 12]. A knee flexion angle of 20 to 30° is recommended for optimal evaluation, as this range ensures consistent assessment of the patellar position relative to the femoral trochlea and minimizes bony overlap [2, 3, 5]. Excessive knee flexion should be avoided, as it may mask patellar subluxations or be detrimental in knees with fractures [2, 3, 5]. Previous studies have primarily focused on compensatory methods for deviations in standard radiographic techniques, particularly in knee anteroposterior and lateral views [13–15]. However, these studies have not addressed skyline views. A study by Nord et al. pointed out inconsistencies in radiographic protocols for knee axial images, emphasizing the need to document knee flexion angles and radiographic techniques on the radiographs [4]. Our study contributes to this body of research by investigating the impact of BPA errors on the accuracy of patellofemoral index measurements. We observed that patellofemoral indices can still be reliably measured, even in radiographs with overlapping articular surfaces and indistinct borderlines due to BPA deviations.

Regarding the consistency and reliability of patellofemoral index measurements, studies have yielded mixed results [16–20]. A meta-analysis by Smith et al. showed a reasonable level of inter-observer and intra-observer reliability for the sulcus angle [18]. However, it indicated insufficient evidence for the reliability of other measurements, such as the congruence angle and patellar tilt, primarily due to unclear details in positioning and radiographic techniques [18]. A meta-analysis by White et al. revealed that the sulcus angle is the only trochlear morphology measure with substantial reliability data, demonstrating both intra- and interobserver reliabilities exceeding 0.75 [21]. Another study by E et al. showed that both the intra- and interobserver ICCs for the sulcus angle, congruence angle, and lateral patellar tilt were over 0.8 for manual measurements. Additionally, they developed a deep learning-based automatic measurement system, with a performance comparable to that of radiologists [22]. The strong ICCs observed in our study, alongside the allowance for a 5° BPA deviation, suggest that our findings could contribute to minimizing the necessity for repeated radiographs.

The use of the anterior border of the proximal tibia as a reference for BPA was adopted from its clinical use as a relatively consistent landmark for determining the tibial mechanical axis, particularly in surgical procedures [10, 23]. Previous studies have often lacked detailed descriptions of precise measurements, primarily focusing on the position and beam angle, but not specifying the methodology for accurately shooting the BPA relative to the knee. Determining this angle accurately can be particularly challenging in obese patients or those with significant tibial bowing. To address this, our study utilized the anterior border of the proximal tibia, defined as the line connecting the tip of the tibial tuberosity to the proximal third of the tibial cortex, as a guide for aligning with the patellar articular surface. This method resulted in an average deviation of 1.5° from the patellar articular surface, a discrepancy deemed clinically acceptable, given the difficulty in discerning a deviation of 1.5° through visual inspection by radiographers. Furthermore, the significance of this observation is further accentuated by our study’s findings, which indicate that patellofemoral indices demonstrate consistent measurements even amidst variations in BPAs. This substantiates the anterior tibial cortex as a lenient, yet reliable, landmark in skyline view.

## Limitations

This study has several limitations. First, our analysis was focused on the Laurin view, which may limit the applicability of our findings to other radiographic views, such as the Merchant or Hughston views, and to patients with more pronounced flexion contractures. Second, the simulation study primarily involved older patients with severe osteoarthritis scheduled for knee arthroplasty, introducing a potential for selection bias. Third, the lateral facet angle and bisect ratio were not measured in the proof of concept study due to the inherent limitations of plain radiographs. Additionally, deviations in BPA exceeding 5° were not examined because of increased bony overlap and decreased resolution. In clinical practice, images with such deviations are typically retaken to achieve a clearer view. Furthermore, exposing real patients to additional radiographic testing for these angles would be unethical due to increased radiation exposure. Lastly, the study did not systematically assess potential sources of error, including maintaining the precise knee flexion angle, the manual positioning of the detector held by the patient, and the accuracy of the beam projection itself.

In summary, the patellofemoral indices in the skyline view remained consistent regardless of BPA deviations. The anterior border of the proximal tibia proved to be an effective landmark for accurate beam projection.

## Data Availability

Not applicable.
